# The Interactions of Soy Protein and Wheat Gluten for the Development of Meat-like Fibrous Structure

**DOI:** 10.3390/molecules28217431

**Published:** 2023-11-04

**Authors:** Yu Peng, Dandan Zhao, Mo Li, Xin Wen, Yuanying Ni

**Affiliations:** 1 College of Food Science and Nutritional Engineering, China Agricultural University, No. 17 Qinghua East Road, Beijing 100083, China; yu1.peng@outlook.com (Y.P.); limo_0125@163.com (M.L.); nyy@cau.edu.cn (Y.N.); 2 College of Food Science & Biology, Hebei University of Science and Technology, No. 26 Yuxiang Street, Shijiazhuang 050000, China; zdd6364@126.com

**Keywords:** soybean protein, wheat gluten, meat analogue, fibrous structure, functional properties

## Abstract

Consumers who are environmentally and health conscious are increasingly looking for plant-based alternatives to replace animal-based products in their daily diets. Among these alternatives, there is a growing demand for meat analogues that closely resemble the taste and texture of meat. As a result, significant efforts have been dedicated to developing meat analogues with a desirable meat-like structure. Currently, soy protein and wheat gluten are the main ingredients used for producing these meat analogues due to their availability and unique functionalities. This study observed that high moisture extrusion at moisture levels of 50–80% has become a common approach for creating fibrous structures, with soy protein and wheat gluten being considered incompatible proteins. After the structuring process, they form two-phase filled gels, with wheat gluten acting as the continuous phase and soy protein serving as a filler material. Moreover, the formation of soy protein and wheat gluten networks relies on a combination of covalent and non-covalent interaction bonds, including hydrogen bonds that stabilize the protein networks, hydrophobic interactions governing protein chain associations during thermo-mechanical processes, and disulfide bonds that potentially contribute to fibrous structure formation. This review provides case studies and examples that demonstrate how specific processing conditions can improve the overall structure, aiming to serve as a valuable reference for further research and the advancement of fibrous structures.

## 1. Introduction

Plant-based diets have gained global attention for their positive impact on both environmental sustainability and health benefits, resulting in a growing trend of replacing animal-based products with plant-based products [1,2]. A significant example of this shift can be seen in the market share of soy protein, which was previously dominated by its usage in animal feed. However, in 2022, the gap between its usage in animal feed and in food and beverages has nearly disappeared globally [3]. Notably, North America is the dominant player in the soy protein market, and the majority of its applications are found in food and beverages. Specifically, its market demand is led by meat and dairy alternatives, accounting for a volume share of 44% in 2022 [3].

The soy protein industry has undergone significant development, with substantial investments made throughout its value chain from farm to fork, ensuring a cost-effective price for soy protein ingredients [4]. Although anti-nutritional compounds, such as trypsin inhibitors, initially hinder the acceptance of soy by impeding pancreatic trypsin and chymotrypsin action in the gut, leading to various disorders, thermal treatment in food processing has been found to effectively reduce or eliminate the negative effects of these anti-nutritional factors in soy protein ingredients [5,6]. Moreover, soy protein has one of the highest scores for digestibility and has a well-balanced amino acid composition, making it a versatile ingredient with a neutral taste profile [7,8]. Furthermore, soy has nitrogen-fixing properties [9], and the production of soy protein for direct human consumption is associated with reduced deforestation and comparatively lower environmental impacts. This is because it requires significantly less land and emits far fewer greenhouse gases compared to its use as animal feed for livestock husbandry [7,10,11,12]. Overall, all these attributes position soy protein as the most valuable alternative to animal protein options, and soy protein continues to be the primary ingredient in most meat analogue products worldwide. In fact, the meat analogue sub-segment accounts for approximately 22% of the global soy protein market [3,13].

The fibrous structure is a crucial element in ensuring consumer acceptance of meat analogue products [14,15]. This is why wheat gluten, alongside soy protein, plays a vital role in developing the desired texture. Wheat gluten possesses a distinctive capability to form thin protein films upon elongation, making it ideal for creating fibrous protein-based materials [16]. Moreover, its functional properties make it suitable as both a binding agent and a structuring agent in the formulation [17,18,19]. An additional advantage is its cost-effectiveness for the industry, as wheat gluten is obtained as a by-product of wheat starch production [20]. The extraction process of gluten from wheat involves only water to wash out the soluble and dispersible components, leaving behind the insoluble proteins [21].

In this overview, we provide an initial introduction to soy protein and wheat gluten, which act as the main ingredients in plant-based meat analogues. Then, we delve into the discussion of advanced technologies used for creating the desired structure. Emphasis is placed on protein interactions and the potential underlying mechanisms behind fibrous structure formation. Furthermore, we elaborate on different formulations for the development of meat analogues and present case studies and examples that highlight how specific processing conditions can enhance the overall structure.

## 2. Protein Ingredients

### 2.1. Soy Protein

Soybeans contain both water-soluble and insoluble proteins, which can be separated into storage globulin and whey fractions by acidification to an approximate pH of 4.5 [22]. The whole extractable globular proteins present in aqueous solutions can be categorized into four main groups: 2S, 7S, 11S, and 15S. These categories have different sedimentation properties under centrifugal force, in which the 7S (β-conglycinin) and 11S (glycinin) fractions account for over 80% of the proteins [23]. The functionalities of soy protein ingredients are determined by several factors, including fractionation pathways, protein content, protein composition, and the presence of other compounds such as oil and carbohydrates [24,25,26].

**Figure 1:**
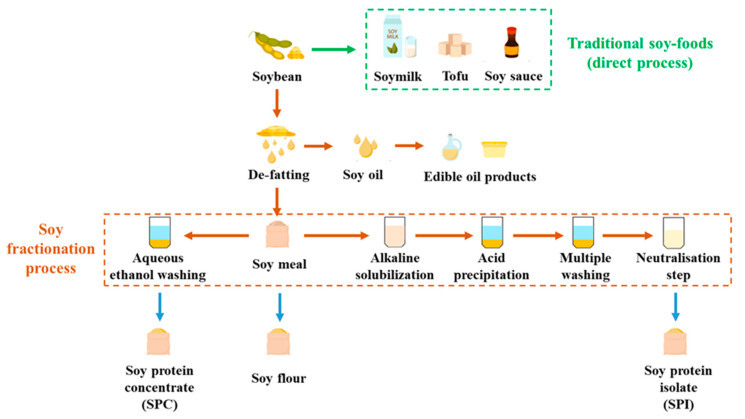
Flow chart of soy protein fractionations.

Soy protein ingredients, such as soy protein concentrate (SPC) and soy protein isolate (SPI), are broadly used in various industries. SPC typically contains around 65% protein, while SPI contains at least 85% protein. There are two common pathways for fractionating SPC and SPI (Figure 1). To produce SPC, de-fatted soy meal (DFSM) is washed with hot water, acidic solution, or aqueous ethanol, with the latter being the most industrially used approach [27]. During this washing process, the soy protein is retained in the solid phase along with the insoluble carbohydrates such as fiber, while the soluble carbohydrates are removed with the solvent. On the other hand, the fractionation of SPI involves an alkaline solubilization step. The protein in the DFSM is extracted in the liquid phase, while the insoluble carbohydrates are separated and discarded in the solid phase [28]. This step aims to achieve high protein solubility for maximum protein extraction and increased yield. The soluble carbohydrates are further eliminated during protein precipitation. Consequently, SPI is considered the most refined soy protein ingredient with the highest protein purity.

Soy protein ingredients were developed to enhance the economic feasibility of soy refining [29]. They have been widely utilized as functional ingredients or cost-effective substitutes in various products. For instance, SPC is commonly added to processed meat, fish, and poultry items to enhance their color and flavor. SPI, with its high viscosity and solubility, finds application in soups, sauces, and beverages. Additionally, SPI with its forming and whipping characteristics can effectively replace egg whites, reducing ingredient costs [30]. In all of the mentioned food products, the required level of soy protein ingredients to achieve the desired effects is low, without introducing negative attributes such as off-flavors, odor problems, or unwelcome textures. Consequently, current soy fractionation processes are optimized to attain high protein purity and specific functionalities, including increased solubility, water absorption, and viscosity, in order to maximize functionality while minimizing adverse effects [31]. However, the growing popularity of plant-based products like meat analogues imposes new requirements for soy protein ingredient purity and functionality.

### 2.2. Wheat Gluten

**Figure 2:**
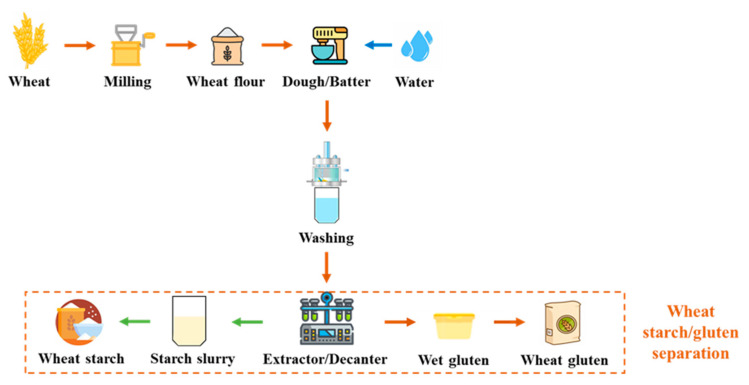
Flow chart of wheat starch/gluten separation.

The approach of separating wheat flour into starch and gluten has been understood for nearly 300 years. However, it was only during the second half of the 20th century that wheat gluten began to be traded as a commodity, eventually gaining recognition as a valuable vegetable protein (Figure 2) [32]. Currently, it is widely employed as a food additive to enhance flour for bread production, and as an ingredient in various food and non-food systems. Its distinctive characteristics, including its capability to form an elastic mass when mixed with water, its ability to retain water, and its thermosetting properties, make it well-suited for a diverse range of applications [33].

Wheat gluten is a cohesive and viscoelastic proteinaceous substance obtained as the by-product of wheat starch production. Gluten protein constitutes approximately 85% of all wheat proteins that are deposited in the wheat endosperm cells, and consists of a diverse mixture of proteins. About half of these proteins are monomeric gliadins, while the remaining portion forms the polymeric glutenin fraction through disulfide cross-linking [34]. Gliadins can be extracted using aqueous ethanol, and have a molecular weight ranging from 30,000 to 60,000. When cysteine residues are present, they are involved in intrachain disulfide bonds [35,36]. Glutenins, on the other hand, are large polymers with a molecular weight of up to twenty million, and are composed of GS units linked by disulfide bonds. Within the GS units, there are high molecular weight (HMW 70,000 to 90,000) and low molecular weight (LMW 30,000 to 45,000) variants [37]. Unlike gliadins, GS units are capable of forming interchain disulfide bonds even at room temperature. When heated, glutenin and gliadin undergo cross-linking, due to oxidation of free sulfhydryl groups, and interchange reactions involving disulfide bonds, resulting in the formation of a significant protein network. Additionally, the formation of disulfide bonds can be regulated by introducing redox agents [38].

Gluten has an extensible nature and can be stretched and pulled, which corresponds with its unique film-forming properties. The elastic properties of gluten can be attributed to glutenin, while gliadin contributes to the viscosity of the protein network [39]. Glutenin exhibits high strength but limited elongation, whereas gliadin enhances extensibility but compromises water stability in gluten films [33]. Previous research has demonstrated that wheat gluten, when subjected to high moisture extrusion or shear structuring as a single component, forms protein gels with anisotropic structures [18,40]. Additionally, wheat gluten facilitates the creation of thin protein films through deformation and elongation, transforming meat analogue dough into a three-dimensional fibrous structure [41]. The water absorption and swelling properties of gluten further contribute to reducing cooking losses during processing and enhancing slicing characteristics [15]. Hence, wheat gluten has been used as key matrix material in the formulation and production of meat analogue products with a fibrous structure.

Soy protein and wheat gluten are plant proteins with distinct fractionation approaches, protein profiles, properties, and functionalities. However, they fulfill the requirements for nutritional and functional characteristics, and act as bulk ingredients in the development of meat alternatives. In order to achieve the desired texture and appearance in current meat analogues, the selection of appropriate protein-rich ingredients is important; furthermore, the utilization of established texturization methods also play a crucial role in creating meat-like structures.

## 3. Structure Formation

Various technologies have been studied and developed for creating a meat-like fibrous structure using soy protein and wheat gluten, including low/high moisture extrusion (LME/HME), spinning, shear cell, and 3D printing. HME is the most commonly used technology in the industry, while the others are still in the developing stage at laboratories. Table 1 summarizes some of the formulations and processing conditions that are soy protein- and wheat gluten-based for the development of meat analogue products.

### 3.1. Low/High Moisture Extrusion

Extrusion is a popular method used in the production of protein-based foods, and it was first introduced in the 1970s to create soy-based meat substitutes; the obtained products appear as a relatively flat, elongated, and longitudinally aligned fibrous mass that closely resembles meat in terms of compactness and chewiness [59]. Currently, these texturized proteins are commercially manufactured using two extrusion methods: low moisture extrusion (LME) and high moisture extrusion (HME) [60].

**Figure 3:**
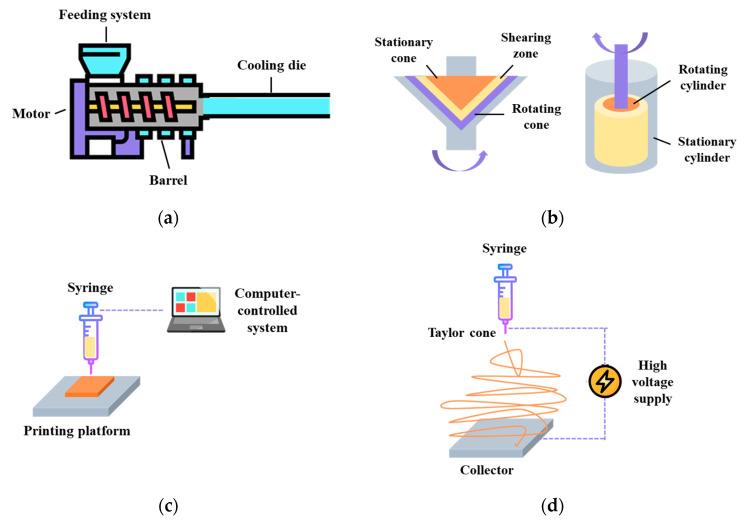
Current structuring technologies for meat-like fibrous structure formation. (**a**) Extrusion, (**b**) shear cell and Couette cell, (**c**) 3D printing, and (**d**) protein spinning.

The process of creating meat analogues through extrusion Involves three main steps: preconditioning, mixing/cooking, and cooling. This process typically requires a setup that includes a preconditioning system, a feeding system, a screw, a heated barrel equipped with a single screw or twin screw, and a cooling die (Figure 3a) [61]. In the case of LME, protein ingredients and other additives are mixed under low moisture conditions. The shaping of the mixture is achieved through thermo-mechanical means, making the preconditioning and cooling steps less crucial in this process [62]. The end products produced through LME have a sponge-like structure, and require rehydration before cooking or frying. Since they have excellent water absorption properties, they are commonly used as meat extenders in the industry to improve the water-holding capacities of processed meat products like burger patties and sausages [63]. The HME technology, developed in the late 1980s, is based on the traditional food extrusion process. Unlike the process of LME, HME is primarily used for food mixtures with moisture contents exceeding 50% [64]. As a result, the end products obtained through HME have a relatively higher water content. During the production of meat analogues, fibrous structures are achieved by extrusion cooking of soy protein and wheat gluten at moisture levels ranging from 50 to 80%. This process takes place at barrel temperatures above 140 °C, with the use of long cooling dies [65]. The moisture levels play a crucial role in the HME process, as they reduce or prevent the dissipation of energy and product expansion. However, they also facilitate necessary operations such as protein gelation, fat emulsification, and the restructuring and shaping of protein constituents [66].

The successful production of meat analogue products with a fibrous structure is influenced by various processing conditions, such as the screw diameter, rotor speed, barrel temperatures, and the specific plant proteins and formulations used (Table 1). Several studies have highlighted the significance of processing conditions in extrusion processes, with particular emphasis on the importance of the processing temperatures. It has been reported that the temperature during processing is a critical parameter for achieving fibrillation of soy protein and wheat gluten, as it affects protein–protein interactions [67,68,69,70]. Therefore, there is a need for dedicated efforts to optimize extrusion parameters to meet the specific requirements of each meat analogue product.

### 3.2. Shear Cell

In the early 2000s, shear cell devices were developed for studying the effects of simple shear on the overall properties of biopolymers like starch or proteins [71,72]. Two types of shearing devices were developed over the years: a conical device based on a cone-plate rheometer (shear cell) and a cylindrical device for scaling up (Couette cell). The shear cell device has a stationary top cone and a rotating bottom, while the Couette cell has a stationary outer cylinder and a rotating inner cylinder [63] (Figure 3b). Both the shear cell and HME processes utilize thermo-mechanical forces to generate fibrous structure formations, and they follow similar key stages, which include preconditioning, mixing/cooking, and cooling [73]. Preconditioning can be done manually (lab-scale) or using a Z-blade mixer (pilot-scale), while mixing/cooking can be achieved through circulating heated oil (shear cell) or a steam jacket (Couette cell). The cooling step is achieved by circulating cooled oil with the shear flow stopped [73].

The implementation of the shearing process to induce fibrous hierarchical structures in dense calcium caseinate has led to the recognition of shear cell technology as a potential tool for creating fibrous anisotropic structures for meat analogue development [74]. Evaluations of the impacts of the shearing process on the structure formation of plant-based biopolymers have been conducted. Fibrous-structured samples have been processed using soy protein and wheat gluten mixtures. These mixtures were processed under different conditions and formulations using both the shear cell and Couette cell (Table 1). Typically, a dough is prepared by mixing soy protein and wheat gluten with water and allowing it to hydrate for 30 min at room temperature. The moisture contents used in the shear cell process are also comparable to HME, and range from approximately 50 to 70%. The hydrated dough is then transferred to a pre-heated shearing device to initiate the shearing process [73]. Thermo-mechanical treatment is carried out at a constant shear rate for 15 min while heating, with distinct fibrous structures predominantly forming at a processing temperature of 140 °C for soy protein–wheat gluten blends [75]. The blends are subsequently cooled and taken out once the temperature is lower than 50 °C, a temperature level that effectively solidifies the material and prevents expansion caused by water evaporation during device opening.

Apart from soy protein and wheat gluten systems, shear cell technology has successfully created a meat-like fibrous structure using a greater variety of plant-based materials [52,76,77,78]. Additionally, research has indicated that the mechanical energy input required for the structuring process can be significantly lower compared to forced assembly processes like extrusion [79]. While shear cell technology is predominantly conducted in small-scale laboratory setups and typically involves prolonged exposure to shear forces for the desired structural changes, the design of the shear cell allows for increased productivity of meat analogues [63]. This can be achieved by increasing the capacity of the device through adjustments to cylinder size, length, or product thickness. However, further studies are necessary to comprehend the underlying principles and complexities of shear flow, and its impact on material structuring. Overall, shear cell technology holds significant potential for scaling up the industrial production of meat analogues.

### 3.3. 3D Printing

3D printing, initially developed in the 1980s for material science applications, is a revolutionary digital process that allows for the creation of intricate solid forms [80]. The process begins with the design of a digital template that defines the desired 3D shape. Then, this template guides a digitally-controlled XYZ robotics system, which constructs the item, layer by layer, starting from the bottom and moving upwards [81] (Figure 3c). The layers can be connected during the construction process or through a separate post-construction step. The supplied materials can be classified into liquids, powders, and cultures of cells, while the printing technologies can be grouped into extrusion printing, inkjet printing, binder jetting, and selective sintering printing [57,82]. In recent years, the food industry has shown a keen interest in 3D printing technology due to its multiple advantages, such as the ability to customize food designs, personalize nutrition, simplify the supply chain, and broaden the availability of food materials [83]. In the case of meat analogue development, the fibrous structure formation is being investigated using 3D printing mainly because it can control the distribution of different components easily to achieve a more similar appearance and appealing shapes [84].

Depositing liquid-based materials can be achieved through extrusion and inkjet printing, with extrusion printing being the most commonly used method [85]. Coaxial extrusion is particularly beneficial as it allows for the cross-linking of two materials, resulting in an encapsulated core and shell configuration. This enables the production of complex and re-defined appealing products with desired shapes [84]. Coaxial extrusion 3D printing is ideal for depositing components independently and simultaneously, making it suitable for creating fibrous structures like meat analogues [84]. Initially, SPI-based mixtures with carrageenan, xanthan gum, sodium alginate, and gelatin were developed as 3D printing materials [86,87]. Subsequently, soy-based fibrous structures were created using a coaxial nozzle incorporating hydrocolloids [88]. High-protein edible printing materials were further developed by mixing soy protein and wheat gluten powders. To create meat analogue products, composite gels were 3D-printed with the facilitation of thermos-sensitive cocoa butter and rice protein [56,57,89].

3D printing technology offers the advantage of creating intricate and visually appealing structures in edible products, thus enhancing consumer interest and appetite [83]. In theory, this technology can be utilized to produce fibrous and anisotropic structures that closely resemble meat muscular tissues. Furthermore, the composition of meat analogue products, including the ratios of soy protein/wheat gluten, moisture content, and types of fat, can be easily adjusted to customize the texture, taste, and flavor according to individual preferences [88]. However, research on 3D-printed plant-based meat analogues is currently limited. To achieve precise and accurate printing of meat-like fibrous structures, several factors must be considered, such as the functionalities of plant protein ingredients, process parameters, and post-processing methods. Although some attempts have been made in producing meat analogues using 3D printing technology, their widespread application remains limited. Therefore, more efforts should be dedicated to the precise and efficient printing of fibrous structures, with the aim of maximizing the technical and commercial potential of 3D printing technology in creating exceptional meat analogue products.

### 3.4. Protein Spinning

Spinning, a well-established technique, was first observed in the early 1900s and patented in the 1930s [90,91]. Wet spinning and electrospinning have been extensively studied for the formation of fibrous structures, with a key distinction in the alignment of biopolymers. During the wet spinning process, a viscous polymeric solution is forced through a spinneret, leading to stretched and aligned fibers. These fibers are subsequently solidified in a coagulation bath containing salts, acids, or alkalis, requiring a vital washing step and generating large waste streams. Consequently, a more appealing alternative method that has gained attention is electrospinning [63,92]. Electrospinning is a technique that employs electrical forces to create polymer fibers, ranging in diameter from 2 nm to several micrometers. The basic components required for an electrospinning apparatus include a high-power voltage supply, a capillary tube with a needle or pipette, and a collector or target (Figure 3d). This process allows for the production of unique natural nanofibers and fabrics with a controllable pore structure, offering great potential for various applications [93,94].

The wet spinning process of proteins presents a challenge as most proteins cannot be spun under food-grade conditions. In the case of the electrospinning process, it has been investigated to structure proteins on a nanometer-length scale, and it is able to act as a means to produce thin fibrils that can serve as textural building blocks for meat analogue products [95]. Due to the complex secondary and tertiary structures, plant proteins pose a considerable challenge when it comes to electrospinning. To successfully electrospin proteins or protein blends with other polymers, the solution must meet certain requirements, for example, high solubility, conductivity, viscosity, and surface tension [92]. Accordingly, SPI that possesses high solubility and viscosity could potentially be used as an electrospinning material. Moreover, solvents with good solvent quality can disrupt protein interactions, leading to the solubilization of the resulting unstructured protein. By employing this approach, plant proteins can possibly become suitable for electrospinning [95]. Although the utilization of wheat gluten and soy protein–wheat gluten blends for fibrous structure formation by electrospinning process has not yet been reported, zein has been successfully spun after solubilization in 70% ethanol [96]. Consequently, further research is necessary to investigate electrospinnable plant proteins and align the fibers for the creation of plant-based meat analogues with a defined structure.

Despite significant advancements in the technology used to structure protein-rich ingredients, the impact of structuring processes on the physical and covalent interactions between proteins remains a topic of extensive research. This complexity arises from the intricate chemical bonding responsible for protein structure. By investigating the interactions between proteins during the structuring process, we can gain valuable insights into the nature of protein–protein bonding.

## 4. Protein–Protein Interactions

Gaining a comprehensive understanding of the interactions between soy protein and wheat gluten, as well as their interactions with other components in the system, could greatly contribute to the comprehension of the underlying mechanisms involved in the formation of fibrous structures during the development of meat analogue products. This knowledge has the potential to resolve discrepancies found in existing literature, enhance production techniques, and ultimately elevate the quality of the end products.

**Figure 4:**
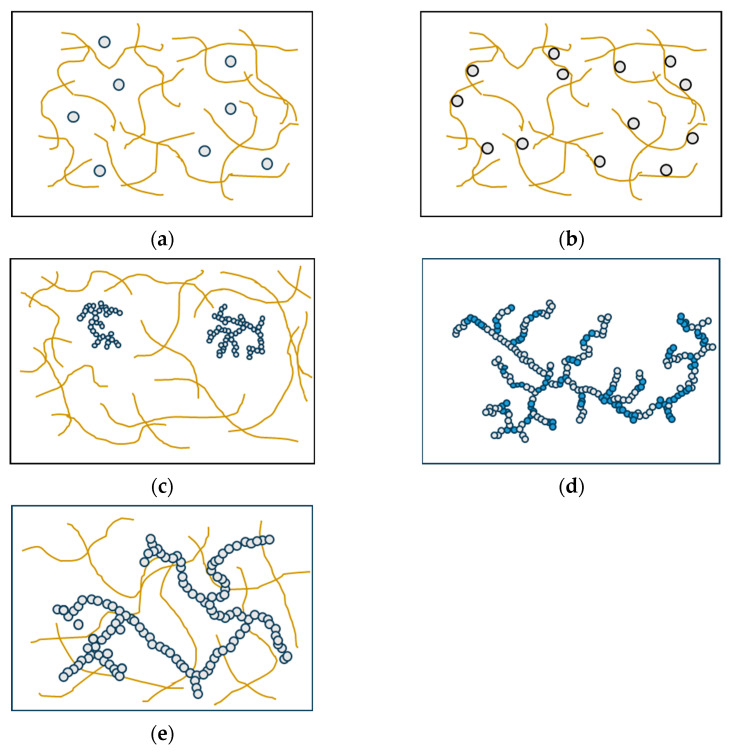
Types of mixed gels. (**a**) Single-phase gels, (**b**) two-phase gels, (**c**) complex gels formed by a non-gelling protein, (**d**) complex gels formed by the copolymerization of two or more proteins, and (**e**) interpenetrating protein gels [96].

Various factors influence the structural formation of multi-component gels through the interaction of two or more proteins. These factors include the proteins’ thermodynamic compatibility, their potential for interaction, and their individual mechanisms of gelation. When different types of protein are mixed, they can be classified as incompatible, semi-compatible, or compatible, depending on whether they form two or more immiscible phases, partially mix at the molecular level, or form a single thermodynamically stable phase [97]. The classification of mixed gels into five distinct types is determined based on the specific functionalities exhibited by individual proteins and the interactions that occur among proteins within the gel system. Filled gels can be formed by incorporating additional protein components into the primary protein gel network. Depending on the phase state of the system, two types of filled gels can be distinguished: single-phase gels, where the filler remains soluble (Figure 4a), and two-phase gels, where phase separation occurs due to thermodynamic incompatibility (Figure 4b) [98,99]. Complex gels are formed when the proteins interact and physically associate with each other. This association can happen randomly through non-specific interactions, where a non-gelling protein reduces the flexibility of the primary network chains and increases the rigidity of the gel (Figure 4c). In this case, the non-gelling protein can be the protein with gelation properties, but the concentration in the mixture is below its least gelling concentration [100]. Another way complex gels form is through the copolymerization of two or more proteins, resulting in a single, heterogeneous network (Figure 4d) [101]. A unique type of multi-component gel is the interpenetrating protein network, in which both protein networks are continuous throughout the gel system (Figure 4e) [97].

Wheat gluten has the ability to form fibrous structures independently after undergoing thermo-mechanical processes such as extrusion and shear cell, and fibrous formations were still observed with the addition of up to 50% soy protein [19,75]. Hence, it can be inferred that soy protein and wheat gluten are incompatible, and the combination of soy protein and wheat gluten falls under the classification of two-phase filled gels, with wheat gluten acting as the continuous phase while soy protein functions as a filler material. The analysis of protein gels developed in a shear cell, using small-angle neutron and X-ray scattering, yielded valuable insights into the micro- and nano-scale structure of flowing two-phase systems [102]. The formation of two phases can align in the shear flow direction during the structuring process [55,73]. Furthermore, in the SPI–WG system, SPI competes with gluten for hydration and the distribution of water is unevenly spread. A noticeably higher water content was observed in the SPI phase compared to the WG phase; as a result, the volumetric fraction of the SPI phase is larger than its mass fraction, and vice versa for the WG phase. Consequently, the higher water absorption by the SPI phase results in a lower concentration of SPI within that phase, leading it to exist either as dispersed liquid particles or as a secondary gel network. It is worth mentioning that the heating and/or structuring processes did not affect the distribution of water within the SPI–WG blend [55].

**Figure 5:**
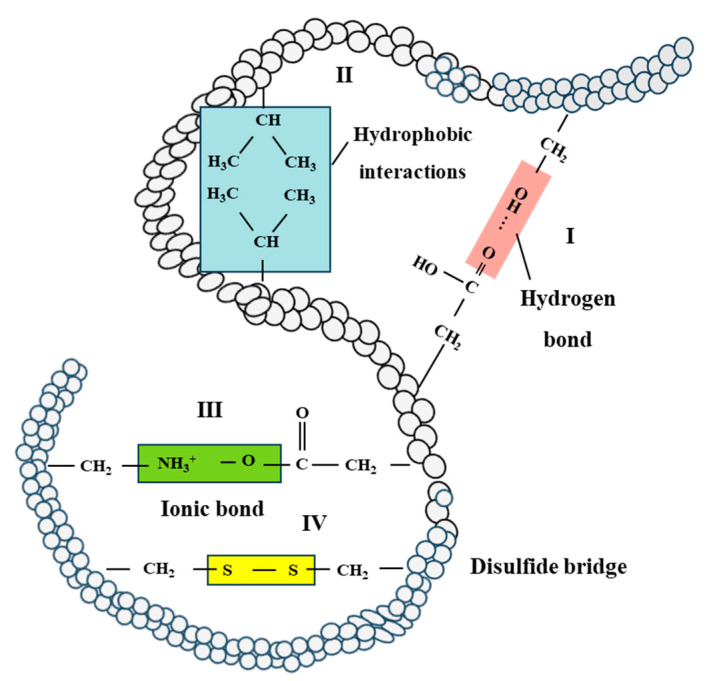
Flow chart of protein–protein interactions in the tertiary structure of protein networks. I: hydrogen bond, II: hydrophobic interactions, III: ionic bond and IV: disulfide bridge [17,34].

The heat-induced gelation of soy protein occurs in four steps: dissociation of small aggregates, association into dense spherical particles, formation of self-similar aggregates, and ultimately, network formation [103]. Hydrophobic interactions are predominant for the formation of SPI gels, while disulfide bonds generally play a role at temperatures over 100 °C (Figure 5) [17,34,104]. Wheat gluten forms a cross-linked network upon hydration, while the formation of disulfide bonds mainly contributes to the heat-induced gluten aggregation and the formation of a three-dimensional network [105]. Furthermore, disulfide bond formations also dominated the wheat gluten polymerization during extrusion, with non-disulfide covalent bonds playing a minor role [19].

The heating and structuring process of soy protein and wheat gluten involves the application of heat and shear, impacting various bonds that stabilize protein structure. As temperature increases, hydrogen bonds are weakened, and hydrophobic interactions can increase [106,107]. Reactive free thiol groups and labile disulfide bonds contribute to thiol-disulfide interchange reactions, forming a transient or reversible network [108,109]. Moreover, shear stress can promote these thiol-disulfide interchange reactions as well [110,111]. Overall, hydrogen bonds, hydrophobic interactions, and disulfide bonds are vital for the formation, stabilization, and retention of fibrous structures in the development of meat analogues. Hydrogen bonds primarily stabilize soy protein and wheat gluten networks, while hydrophobic interactions govern protein chain associations during the thermo-mechanical process, and disulfide bonds may contribute to fiber formation.

## 5. Conclusions

Environmentally and health conscious consumers are increasingly seeking plant-based alternatives to replace animal-based products in their diets, particularly in relation to dairy. The market is seeing a growing demand for meat analogue products that mimic the taste and texture of meat. Currently, the most commonly used plant protein resources for developing these products are soy protein and wheat gluten, mainly due to their availability and specific functionalities. Extensive research has focused on understanding how these proteins interact and form a fibrous structure through various structuring processes. Our findings indicate that both HME and shear cell processes share similar basic processing steps, while 3D-printing and spinning processes exhibit some similarities to a certain extent. Among all of the techniques, extrusion has become the industrially used technique for fibrous structure formation, while other advanced technologies are actively being developed and tested at both the lab-scale and pilot-scale levels. In addition to processing techniques, our research also summarizes formulations and processing conditions and introduces hypotheses regarding the protein–protein interactions during the process. According to our hypothesis, soy protein and wheat gluten generally form two-phase filled gels, and a combination of covalent and non-covalent interaction bonds plays a crucial role in forming soy protein and wheat gluten networks. These bonds include hydrogen bonds, which help to stabilize the networks of proteins, hydrophobic interactions that govern protein chain associations during thermo-mechanical processes, and disulfide bonds that potentially contribute to the formation of fibrous structures.

## 6. Future Directions

In light of the increasing demand for plant-based meat analogue products and the advancements made in understanding protein interactions and structuring processes, future research should focus on several key areas. Firstly, it is necessary to explore different fractionation pathways to produce a wider range of soy protein and wheat gluten ingredients. Currently, their existing fractionation processes are not fully optimized for meat analogue development. By doing so, the applicability and sustainability of plant protein ingredients can be further enhanced. Moreover, more in-depth studies are required to understand the network-forming behavior of soy protein and wheat gluten. The mechanisms behind their formation have not been fully elucidated yet. Gaining a better understanding of these mechanisms would enable precise control over the texture and structure of meat analogue products, ultimately improving consumer acceptance. Furthermore, additional research is needed to optimize the production processes of meat analogues, such as 3D-printing and spinning techniques. Taking a comprehensive approach that incorporates multiple developed technologies will ensure that future developments in meat analogues meet the evolving needs of environmentally and health conscious consumers.

## Figures and Tables

**Table 1:** An overview of soy protein–wheat gluten formulations, techniques, and processing conditions used to produce meat-like fibrous structures.

Formulations	Technique	Processing Conditions	Reference
SPC + WG	HME	Screw length/diameter ratio 44:1, feed rate 7.0 g/min, moisture content 60%.	[42]
SPI + WG	HME	Screw speed 110 rpm, feed speed 5 kg/h, temperature zones at 30, 35, 40, 45, 50, 55, 60 100, 140, and 170 °C along the extrusion direction.	[43]
SPC + WG	HME	Moisture content 57%, maximum barrel temperature 170 °C, dry and water feed rate 2.8 kg/h and 3.6 kg/h, respectively.	[44]
SPI + WG	HME	Screw speed 200 r/min, barrel temperatures 30, 40, 60, 140, 150, and 150 °C sequentially.	[45]
SPI + WG	HME	Feed speed 6 kg/h, feed moisture 55%, cooling die with a length of 164 mm, the end of the extruder barrel 20 °C.	[46]
Soy flour + WG	HME	Screw diameter 20 mm, L/D ratio of 40:1, cooling water temperature 30 °C, flow rate of 6.97 L/min.	[47]
SPI + WG	HME	Moisture content 70%, feed rate 100 g/min, barrel temperatures 100, 160, and 130 °C.	[48]
SP + WG	HME	Length/diameter ratio 15:1, extrusion temperature 170 °C, three moisture levels 72.12, 66.78, and 60.11%.	[49]
SPC + WG	HME	Barrel diameter 11 mm, length/diameter ratio 44:1, feed rate 8 g/min, moisture level 60%, at series temperatures of 40, 60, 80, 100, 120, 150, and 150 °C.	[50]
SPI + WG	Couette Cell	Processing temperature 95 °C for 15 min, shear rate 30 rpm, cooling to 4 °C within 30 min.	[51]
SPI + WG	Shear cell	Processing temperatures of 95–140 °C for 15 min, HTSC cooled down to 25 °C within 5 min.	[52]
SPI + WG	Shear cell	Constant shear rate 30 rpm for 15 min, preheated temperature 95 °C, the samples were cooled to 4 °C within 30 min.	[40]
SPI + WG	Shear cell	Dry matter content 29.4 wt%, pre-heated temperature 95 °C, shear rate 30 rpm for 15 min, cooling to 4 °C within 30 min.	[53]
SPC + WG	Shear cell	Processing temperature 140 °C, shear rate 30 rpm for 15 min, cooling step at 25 °C for 5 min.	[54]
SPI + WG	Shear cell	Processing temperature 140 °C, shear rate 30 rpm for 15 min, cooling step at 25 °C for 5 min.	[55]
SPI + WG	3D printing	3D printing inks were printed at 25 °C after being placed at 4 °C for 12 h.	[56]
SPI + WG	3D printing	Gelatinization temperature 60 °C for 30 min, preheated temperature 60 °C, extrusion distance 5 mm.	[57]
SPC + WG	3D printing	Syringe diameter 22 mm, printing speed 20 mm/s, nozzle diameter 0.8 mm, printing layer height 0.8 mm, printing temperature 25 °C.	[58]
